# Comprehensive analysis for clarifying transcriptomics landscapes of spread through air spaces in lung adenocarcinoma

**DOI:** 10.3389/fgene.2022.900864

**Published:** 2022-08-22

**Authors:** Yuan Zeng, Lingli Zhou, Dexin Jia, Bo Pan, Xiaomei Li, Yan Yu

**Affiliations:** ^1^ Department of Medical Oncology, Harbin Medical University Cancer Hospital, Harbin, China; ^2^ Department of Respiratory Medicine, Suizhou Hospital, Hubei University of Medicine, Hubei, China; ^3^ Department of Pathology, Harbin Medical University Cancer Hospital, Harbin, China

**Keywords:** lung adenocarcinoma, spread through air spaces, differential expression analysis, protein–protein interaction, enrichment analysis, RNA-seq

## Abstract

Patients with spread through air spaces (STAS) have worse postoperative survival and a higher recurrence rate in lung adenocarcinoma, even in the earliest phases of the disease. At present, the molecular pathogenesis of STAS is not well understood. Therefore, to illustrate the underlying pathogenic mechanism of STAS, we accomplished a comprehensive analysis of a microarray dataset of STAS. Differential expression analysis revealed 841 differentially expressed genes (DEGs) between STAS_positive and STAS_negative groups. Additionally, we acquired two hub genes associated with survival. Gene set variation analysis (GSVA) confirmed that the main differential signaling pathways between the two groups were hypoxia VHL targets, PKC, and pyrimidine metabolism pathways. Analysis of immune activity showed that the increased expression of MHC-class-Ⅰ was observed in the STAS_positive group. These findings provided novel insights for a better knowledge of pathogenic mechanisms and potential therapeutic markers for STAS treatment.

## Introduction

Lung adenocarcinoma is the most frequent histologic subtype of lung cancer and has a high risk of recurrence or metastasis at the early disease stage (Shi et al., 2016). It has been proposed that lung cancer metastasis occurs through hematogenous spread, lymphatic spread, and direct infiltration (Han et al., 2021). In 2015, the World Health Organization (WHO) pointed out that spread through air spaces (STAS) is a recently identified pattern of tumor invasion (metastasis) (Travis et al., 2015). STAS is defined as tumor cells within air spaces in the lung parenchyma beyond the edge of the primary tumor (Eguchi et al., 2019; Qi et al., 2021). Notably, several studies have demonstrated that STAS is an independent factor indicating lung cancer recurrence and poor outcomes in patients (Yang et al., 2018; Liu et al., 2020; Onozato et al., 2021). Additionally, STAS is an insidious pattern of invasion that is invisible to pathologists on gross examination and surgeons on external analysis of tumor specimens at the time of surgery. Unfortunately, there is no current, reliable radiological method to detect STAS (Kadota et al., 2015). Therefore, there is a pressing need to investigate the novel potential and pathogenic mechanisms of STAS.

In lung adenocarcinoma, STAS occurrence is related to the interactions between neutrophils and the tumor. The tumor environment releases interleukin-8 to promote the apoptosis of the local tumor neutrophils and activates neutrophils to promote tumor cell shedding from the primary tumor body, allowing them to migrate along the lung basement membrane to another alveolar cavity, resulting in STAS (Wislez et al., 2004; Wislez et al., 2007). With the developments and broad applications of high-throughput technology in biological and biomedical research fields, these tools can monitor genome-wide gene transcription levels and provide insight into biological processes involved in gene regulation (Gerstner et al., 2020; Hess et al., 2020). Therefore, we predicted the pathogenic mechanisms and candidate markers of STAS using high-throughput sequencing.

In our study, we performed an integrative analysis of the gene expression dataset of STAS in lung adenocarcinoma. Firstly, differential expression analysis showed that there were 841 differentially expressed genes (DEGs). Among them, two hub genes, *CXCL8* and *CPB2*, were identified as relevant to survival. Finally, through gene set variation analysis (GSVA), we found that hypoxia VHL targets, PKC, and pyrimidine metabolism pathways were the three main differential signaling pathways. We also observed increased MHC-class-Ⅰ expression in the STAS_positive group by immune activity analysis. In conclusion, the results provided novel insights into the potential biomarkers and underlying molecular mechanisms of STAS in lung adenocarcinoma.

## Materials and methods

### Tissue samples

Tumor samples were obtained from March to July 2021 from 19 lung adenocarcinoma patients who underwent surgical resection at Harbin Medical University Cancer Hospital. The inclusion criteria of this study were as follows: 1) histologically confirmed adenocarcinoma; 2) complete clinicopathological information. Exclusion criteria were as follows: 1) preoperative radiotherapy and/or chemotherapy and 2) tumor with other components, including neuroendocrine or squamous differentiation. The clinicopathological characteristics of samples are shown in Table 1. The research was approved by the Ethics Committees of Harbin Medical University Cancer Hospital; all patients in this trial agreed to participate and signed written consent.

**TABLE 1:** Clinical factors of STAS_positive and STAS_negative groups.

Factors		Overall (*n* = 19)	Positive (*n* = 11)	Negative (*n* = 8)	*p*-value
Age group (years, %)	≥60	11 (57.9)	7 (63.6)	4 (50.0)	0.658
	<60	8 (42.1)	4 (36.4)	4 (50.0)	
Gender (%)	Female	9 (47.4)	4 (36.4)	5 (62.5)	0.37
	Male	10 (52.6)	7 (63.6)	3 (37.5)	
Tumor_size (cm)	Median [IQR]	2.00 [1.35, 3.00]	2.50 [1.50, 3.00]	1.75 [1.15, 2.62]	0.558
N_stage (%)	N0	17 (89.5)	9 (81.8)	8 (100.0)	0.228
	N2	2 (10.5)	2 (18.2)	0 (0.0)	
p stage (%)	IA1	4 (21.1)	2 (18.2)	2 (25.0)	0.454
	IA2	5 (26.3)	2 (18.2)	3 (37.5)	
	IA3	7 (36.8)	5 (45.5)	2 (25.0)	
	IB	1 (5.3)	0 (0.0)	1 (12.5)	
	IIIA	2 (10.5)	2 (18.2)	0 (0.0)	
Smoking (%)	No	11 (57.9)	6 (54.5)	5 (62.5)	1
	Yes	8 (42.1)	5 (45.5)	3 (37.5)	
EGFR (%)	Mutant-type	4 (21.1)	2 (18.2)	2 (25.0)	1
	Wild-type	15 (78.9)	9 (81.8)	6 (75.0)	
ALK (%)	Mutant-type	4 (21.1)	3 (27.3)	1 (12.5)	0.603
	Wild-type	15 (78.9)	8 (72.7)	7 (87.5)	
KRAS (%)	Mutant-type	4 (21.1)	3 (27.3)	1 (12.5)	0.603
	Wild-type	15 (78.9)	8 (72.7)	7 (87.5)	

### Data collection

Clinicopathological characteristics were obtained from medical records, such as age, gender, smoking status, tumor size, N stage, TNM stage, EGFR mutation, KRAS mutation, and EGFR mutation.

**FIGURE 1:**
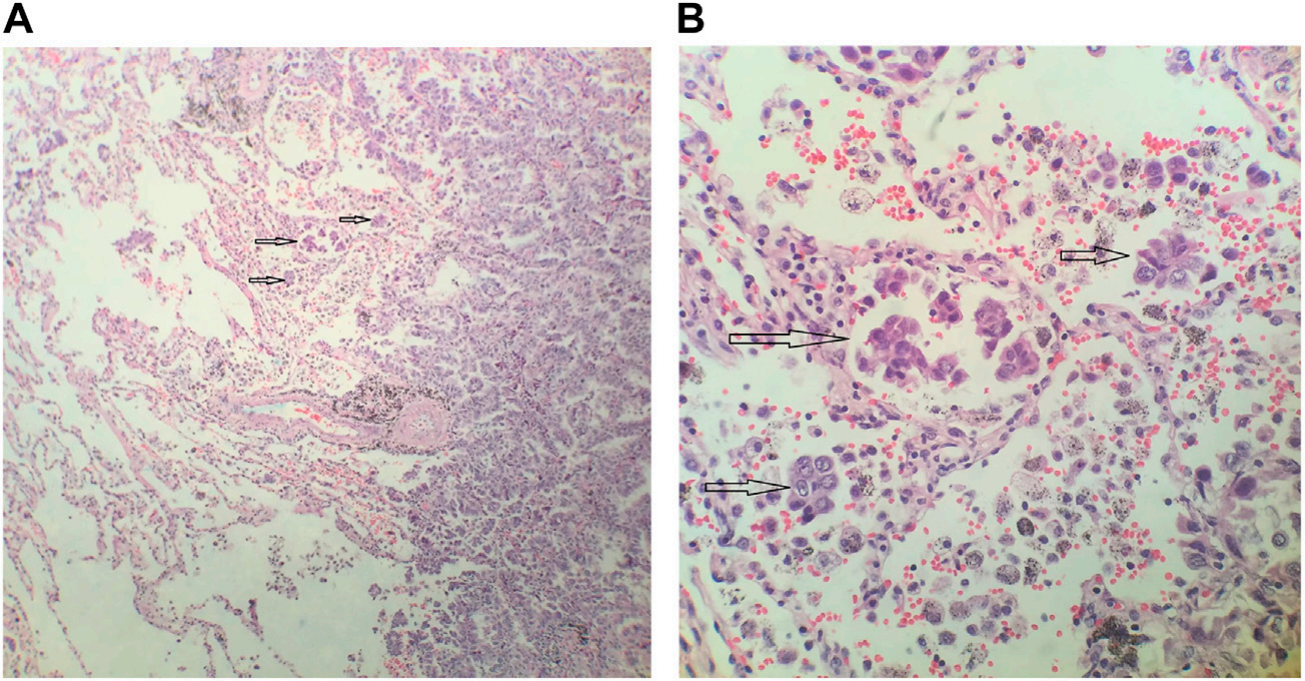
Representative histopathologic images of spread through air spaces (STAS) in lung adenocarcinoma. **(A)** Magnification×100 **(B).** Magnification ×400.

Two pathologists independently inspected all samples, and disagreements were resolved through discussion and consensus. STAS consists of micropapillary clusters, solid nests, or tumor individual tumor cells at the outside edge of the surrounding alveolar lacuna of lung parenchyma. Notably, micropapillary clusters are defined as papillary structures without central fibrovascular cores in the alveolar space. Additionally, solid nests are defined as solid collections of tumor cells filling air spaces. It is well understood that the tumor cells leave the edge of the lung cancer mass and enter the alveoli and bronchioles of the peripheral pulmonary parenchyma. Representative histopathologic images of STAS in lung adenocarcinoma (arrows) are shown in Figures 1A,B. According to the pathological results, samples were divided into the STAS_positive and STAS_negative groups.

### RNA isolation, quality control, library preparation, and sequencing

Total RNA was isolated from paraffin-embedded tissue using the RNeasy Mini kit (Qiagen). The quantity and quality of the extracted total RNA were then determined using a Qubit RNA BR Assay Kit and Agilent 2200 TapeStation. RNA-seq libraries were constructed according to the manufacturer’s instructions using the TruSeq Stranded mRNA Prep kit (Illumina). RNA quality control was verified by applying the MultiNA Microchip Electrophoresis System. Total RNA samples were transferred into 96-well plates and diluted to 1 µg of 50 µl. The purification steps during library preparation were performed using the MinElute PCR Purification kit (Qiagen). After purification and PCR amplification, the final cDNA library was generated according to Illumina’s RNA-seq Library Preparation Protocol. We conducted deep sequencing on the Illumina HiSeq 1500 platform using a TruSeq Rapid SBS kit (Illumina) in a 50-base single-end mode.

### Read preprocessing and gene count normalization

The obtained raw paired-end reads were trimmed using the fastp tool to remove low-quality reads and adapter contaminants. Then, raw read data were aligned to the human reference genome (build 37.2) using the hisat2 (version 2.1.0) tool. DESeq2 and the upper quartile normalization method were used to normalize the raw read counts.

### Identification of differentially expressed genes

FPKM was used to calculate the gene expression level. The DEGs were calculated using the R package DESeq2 (Zhang et al., 2021). p. adj <0.05 and |log2fold change| > 2 were defined as thresholds. The R package ggplot2 was used to construct the Volcano and Heat maps.

### The construction of the protein–protein interaction network

PPI network analysis has emerged as a useful approach to identifying potential new targets and mechanisms from a systematic perspective (Wu et al., 2020). PPI network was constructed using the STRING database (https://string-db.org) (Buttacavoli et al., 2020; Ying et al., 2020). The Cytoscape software was used to analyze the hub genes (Szklarczyk et al., 2017). Log-rank test was used to compare differences in overall survival (OS) and progression-free survival (PFS) between the low- and high-expression groups by median cutoff for each gene. The statistical difference of hub genes between normal and tumor samples was compared through the Wilcox test. All the analysis methods and R package were implemented by R version 4.0.3.

### Gene functional enrichment analysis

Gene Ontology (GO) and Kyoto Encyclopedia of Genes and Genomes (KEGG) analyses of DEGs were carried out using the clusterProfiler package (Version 4.0.3) (Xu et al., 2018). In addition, pathway enrichment analysis of DEGs was conducted using the ReactomePA package (Chow et al., 2020).

### Gene set variation analysis

The gene sets of H (hallmark gene sets), C2 (curated gene sets), and C5 (GO gene sets) were downloaded from the MSigDB database (http://software.broadinstitute.org/gsea/msigdb/index.jsp) for gene set variation analysis (GSVA) (Migliavacca et al., 2019; Park et al., 2017). GSVA was used to identify pathways enriched among expressed genes in two groups. The significant difference was set at a *p-*value <0.05.

### Analysis of factors related to immune microenvironment

We used single-sample gene set enrichment analysis (ssGSEA) to determine the proportions of 28 types of immune cells in the tumor microenvironment (Thorsson et al., 2018). The CIBERSORT software was applied to evaluate the relative abundance of tumor-infiltrating immune and stromal cells (Conley et al., 2021).

The proportions of 28 types of immune cells, the score of immune activity, and the score of tertiary lymphoid structure (TLS) between the two groups were compared and analyzed using the Wilcoxon ranked-sum test.

### CancerSEA

CancerSEA (http://biocc.hrbmu.edu.cn/CancerSEA/) was used to comprehensively investigate the functional status of cancer cells at the single-cell level (Yuan et al., 2019). It could provide information on DEGs in various cancers with multiple functional states.

## Results

### Identification of DEGs between STAS_positive and STAS_negative groups

**FIGURE 2:**
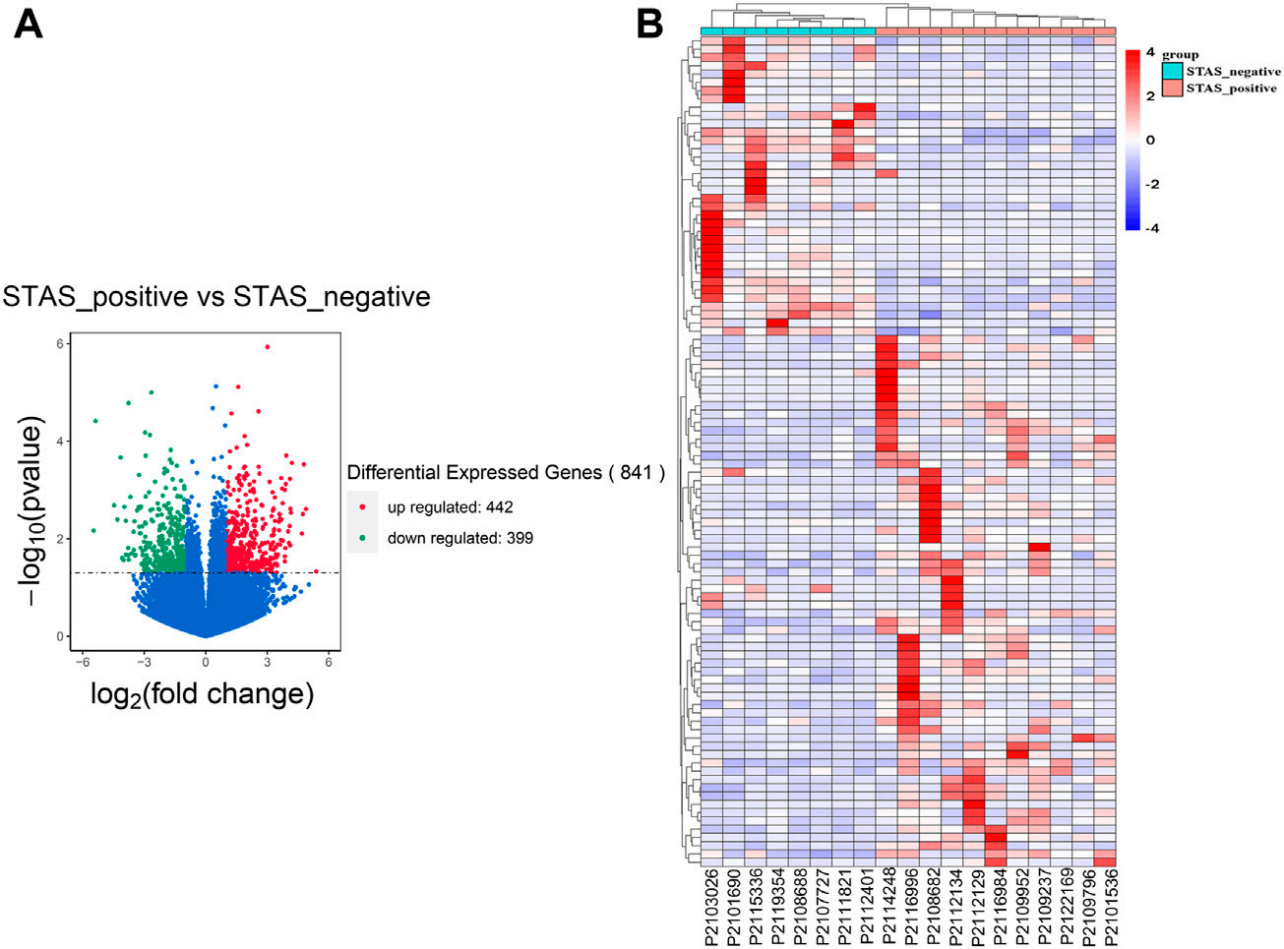
Identification of DEGs between STAS_positive and STAS_negative groups. **(A)** Volcano plot. **(B)** Heat map. The top_100 of 841 DEGs.

The dataset was analyzed for DEGs, as shown in Figure 2A. A total of 841 genes were differentially expressed, with 442 upregulated and 399 downregulated. Moreover, the top_100 DEGs were subjected to cluster analysis. The results showed that the top_100 DEGs could stratify patients in the STAS_positive *versus* STAS_negative groups (Figure 2B).

### Estimation of DEGs on the protein–protein interaction network

**FIGURE 3:**
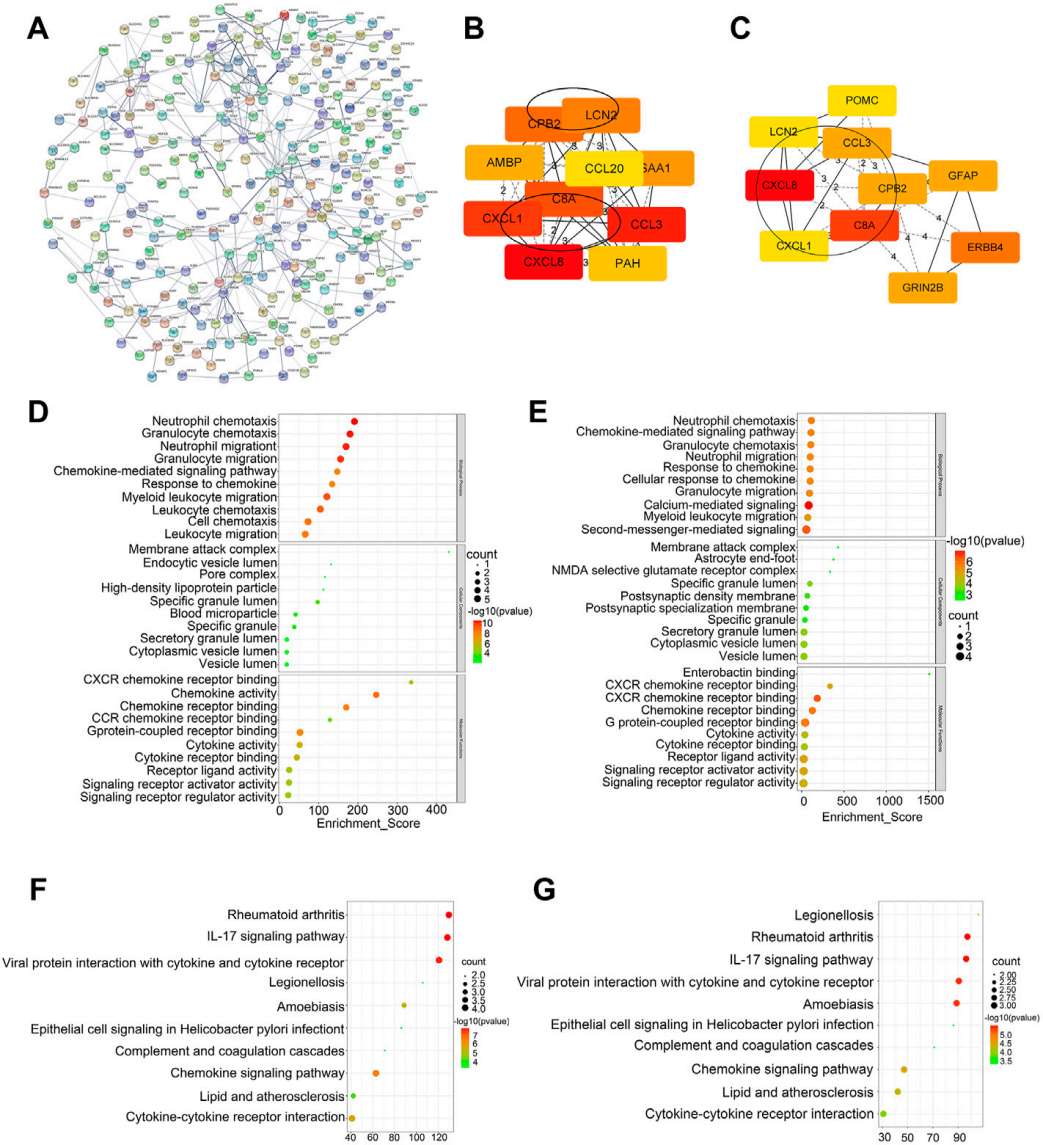
Construction of PPI network and screening of hub genes. **(A)** PPI network of DEGs. **(B)** Top 10 genes ranked by the MCC method. **(C)** Top 10 genes ranked by the MNC method. **(D–E)** GO analysis of top 10 genes ranked by the MCC and MNC method. **(F,G)** KEGG analysis of top 10 genes ranked by the MCC and MNC method.

We performed the PPI network analysis to further investigate the interactions among the 841 DEGs **(** Figure 3A **)**. The results indicated that part of DEGs strongly correlated with other genes. Six hub genes were identified by overlapping the top 10 genes obtained using MCC and MNC ranking methods (Figures 3B,C). The top 10 hub genes ranked by the MCC and MNC methods were functionally annotated through GO terms and KEGG pathways (Figures 3D–G). Based on the GO and KEGG analysis, these hub genes were mainly enriched in cytokine and chemokine-related pathways.

**FIGURE 4:**
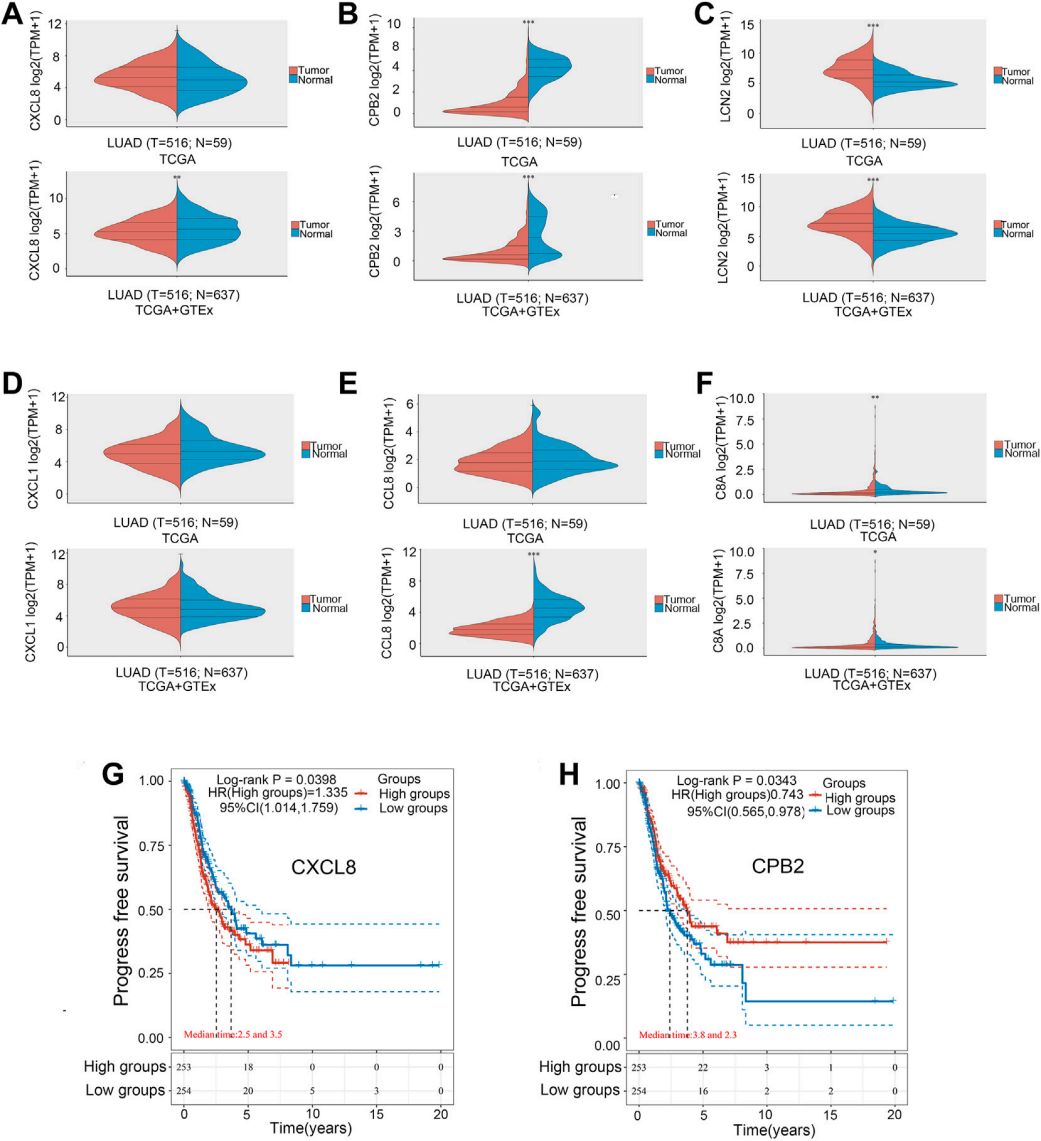
Gene expression levels in the tumor *versus* normal tissue. **(A)** CXCL8. **(B)** CPB2. **(C)** LCN2. **(D)** CXCL1. **(E)** CCL3. **(F)** C8A. Kaplan–Meier survival curve. **(G)** CXCL8. **(H)** CPB2. *p < 0.05, **p < 0.01, ***p < 0.001.

The six hub genes were *CXCL8*, *CXCL1*, *CCL3*, *C8A*, *CPB2*, and *LCN2*. The TCGA and GTE databases revealed that the other five genes significantly differed in gene expression in lung adenocarcinoma tumors *versus* normal tissues, except for the *CXCL1* gene (Figures 4A–F). Clinical data for lung adenocarcinoma were obtained from TCGA. We then performed the OS and PFS analyses of these six hub genes. Figures 4G,H show that the high-CXCL8 expression group had significantly worse PFS than the low-CXCL8 expression group (*p* = 0.0398). It is worth noting that patients with high CPB2 expression had significantly better PFS than those in the low-expression group (*p* = 0.0343). However, no significant differences were found in the other four genes.

### Functional enrichment analysis of DEGs

**FIGURE 5:**
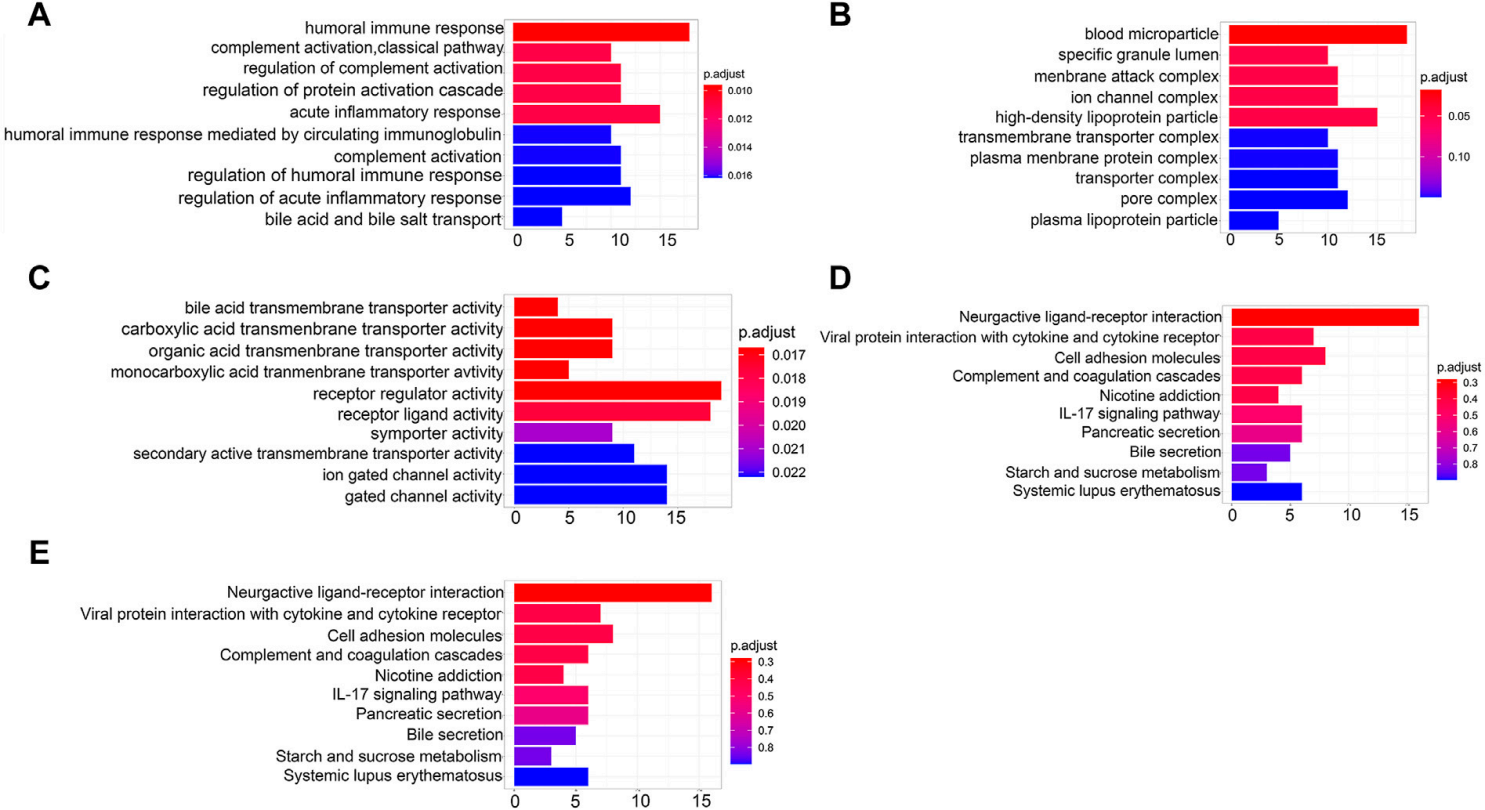
Functional enrichment analysis of DEGs. **(A)** BP analysis of DEGs. **(B)** CC analysis of DEGs. **(C)** MF analysis of DEGs. **(D)** KEGG pathway of DEGs. **(E)** Reactome pathway of DEGs.

GO analysis covers biological process (BP), cellular component (CC), and molecular function (MF). BP enrichment revealed that the identified DEGs were involved in humoral immune response, complement activation, and regulation of protein activation cascade (Figure 5A). CC enrichment revealed that DEGs were involved in blood microparticles, specific granule lumen, and membrane attack complexes (Figure 5B). MF enrichment revealed that DEGs were involved in bile acid transmembrane transporter activity, carboxylic acid transmembrane transporter activity, and organic acid transmembrane transporter activity (Figure 5C). Slight enrichment of GO analysis was observed, but the results were not statistically significant (*p* > 0.05).

DEGs were also analyzed using the KEGG and Reactome pathway enrichment analyses. KEGG pathway analysis showed that the DEGs were enriched in IL-17 signaling and involved in the interaction of neuroactive ligands with the receptors and cell adhesion molecules (Figure 5D). The Reactome pathway analysis indicated that the DEGs were enriched in the transport of organic anions, bile acids, and bile salt metabolism and recycling of bile acids and bile salts signaling pathways (Figure 5E). However, the findings had no statistical significance (*p* > 0.05).

### Evaluation of gene set differences between STAS_positive and STAS_negative groups

**FIGURE 6:**
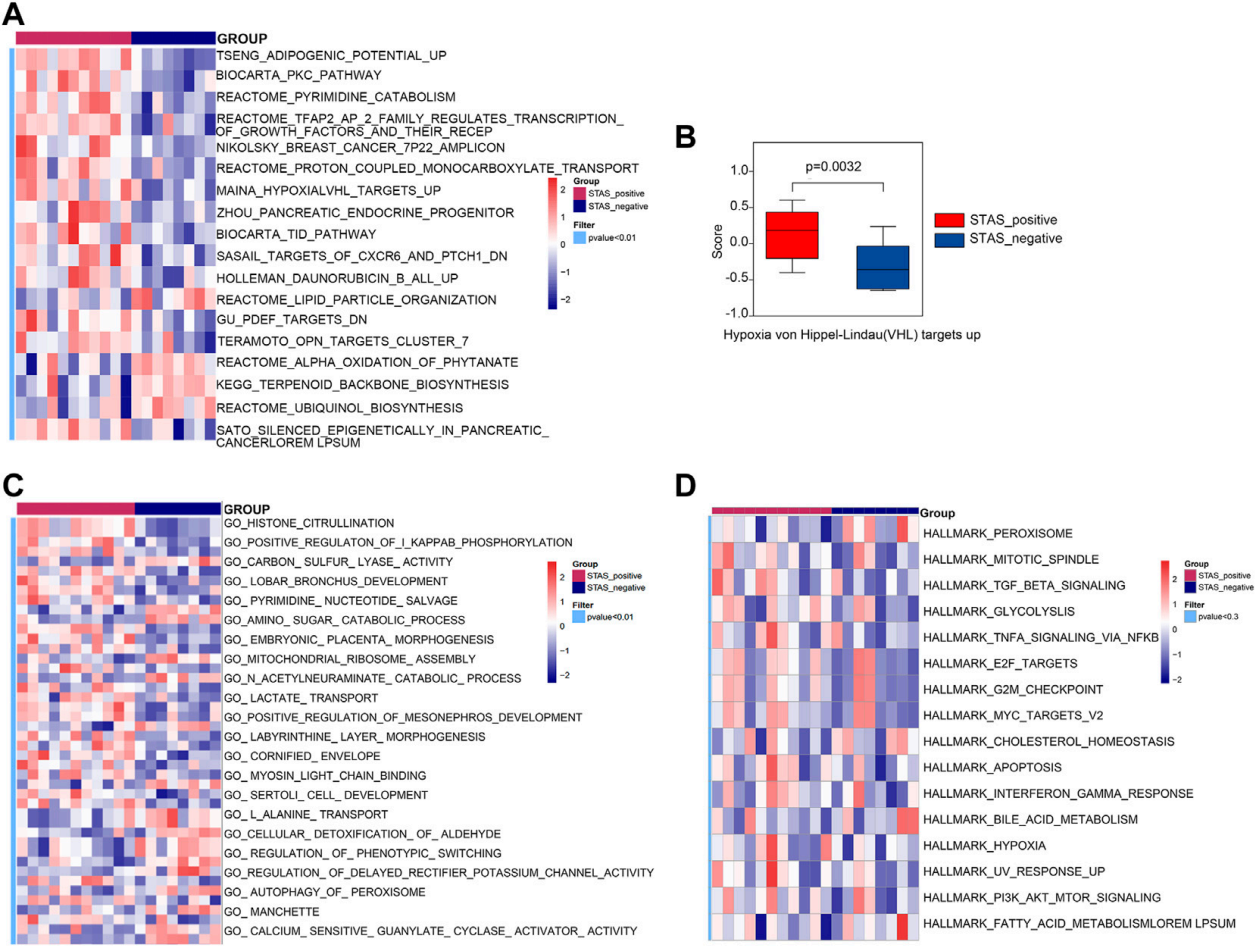
Gene set variation analysis (GSVA) between STAS_positive and STAS_negative groups. **(A)** Heat map derived by C2 gene set. **(B)** Box plot of Hypoxia VHL targets up. **(C)** Heat map derived by C5 gene set. **(D)** Heat map derived by Hallmark gene set.

GSVA was performed using the curated gene sets (C2), GO gene sets (C5), and hallmark gene sets (H). The comparative analysis result using the C2 gene set demonstrated that there were 118 significantly different biological processes and signaling pathways between STAS_positive and STAS_negative groups. Only those biological processes and pathways with *p* < 0.01 are shown in Figure 6A. The main differential signaling pathways included hypoxia VHL targets, PKC, and pyrimidine metabolism pathways. Figure 6B shows a boxplot of hypoxia VHL targets.

The analysis result using the C5 gene set indicated that 247 biological processes and signaling pathways significantly differed between the two groups. Figure 6C only considered biological processes and pathways with *p* < 0.01. The differential biological processes were mainly involved in histone citrullination, protein arginine deiminase activity, and positive regulation of Ⅰ-kappaB phosphorylation.

The analysis result using the Hallmark gene set revealed that 50 signaling pathways differed in score values between the two groups, but the differences were not statistically significant (*p* > 0.05). Figure 6D shows 16 signaling pathways with *p* < 0.3, such as TGF-β, MYC_TARGETS_V2, IFN-γ response, and other signaling pathways.

### Integrative analysis of immune-related factors between STAS_positive and STAS_negative groups

**FIGURE 7:**
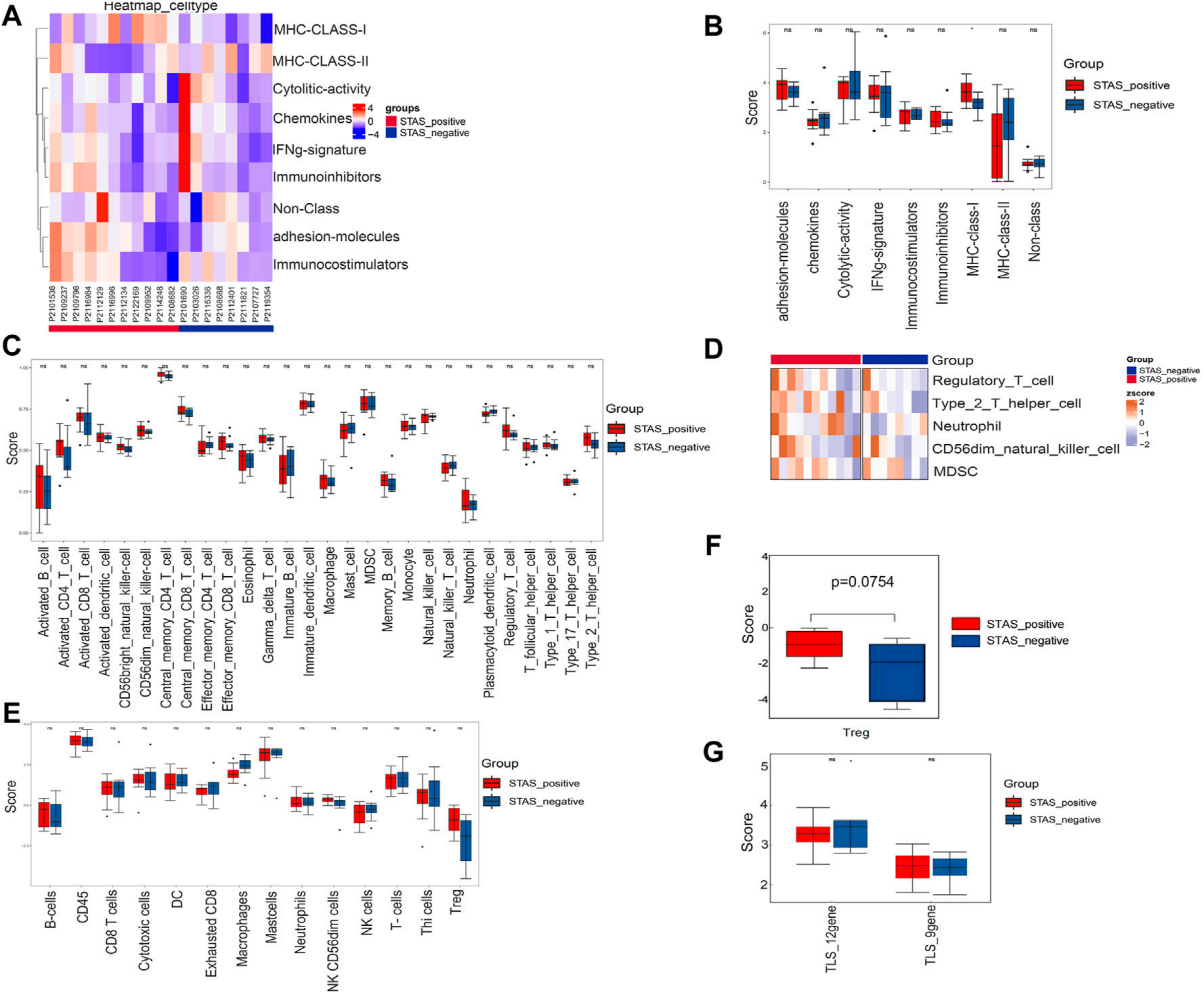
Analysis of related factors of immune microenvironment between STAS_positive and STAS_negative groups. **(A)** Heat map of immunoactivity analysis. **(B)** Box plot of immunoactivity analysis. **(C)** Box plot of proportions of 28 types of immune cells. **(D)** Heat map showing comparative analysis among five cell subsets. **(E)** Box plot of the abundance of 14 immune cell populations. **(F)** Box plot of Treg. **(G)** Box plot of comparison of TLS. *p < 0.05.

The signature score of nine immune features of tumor samples was calculated (Figure 7A). We performed a comparative analysis and found that the expression of MHC-Class-Ⅰ in the STAS_positive group was significantly higher than that in the STAS_negative group (Figure 7B).

The proportions of 28 types of immune cells in the tumor microenvironment are shown in Figure 7C. Although not statistically significant, we did observe more regulatory T cells (Tregs), type 2 T helper cells, neutrophils, CD56^dim^ natural killer (NK) cells, and myeloid-derived suppressor cells (MDSC) in the STAS_positive group (Figure 7D).

Figure 7E shows the abundance of 14 immune cell populations. Wilcoxon test analysis indicated that the abundance of Tregs tended to increase in the STAS_positive group (*p* = 0.0754) (Figure 7F).

TLS is an aggregate of immune cells (mainly T cells and B cells). The scores of two TLS signatures were calculated according to the TLS signature-related gene expression levels reported in the studies (Figure 7G). However, no significant differences were found between the two groups (*p* > 0.05).

### Assessment of functional status features in CancerSEA

**FIGURE 8:**
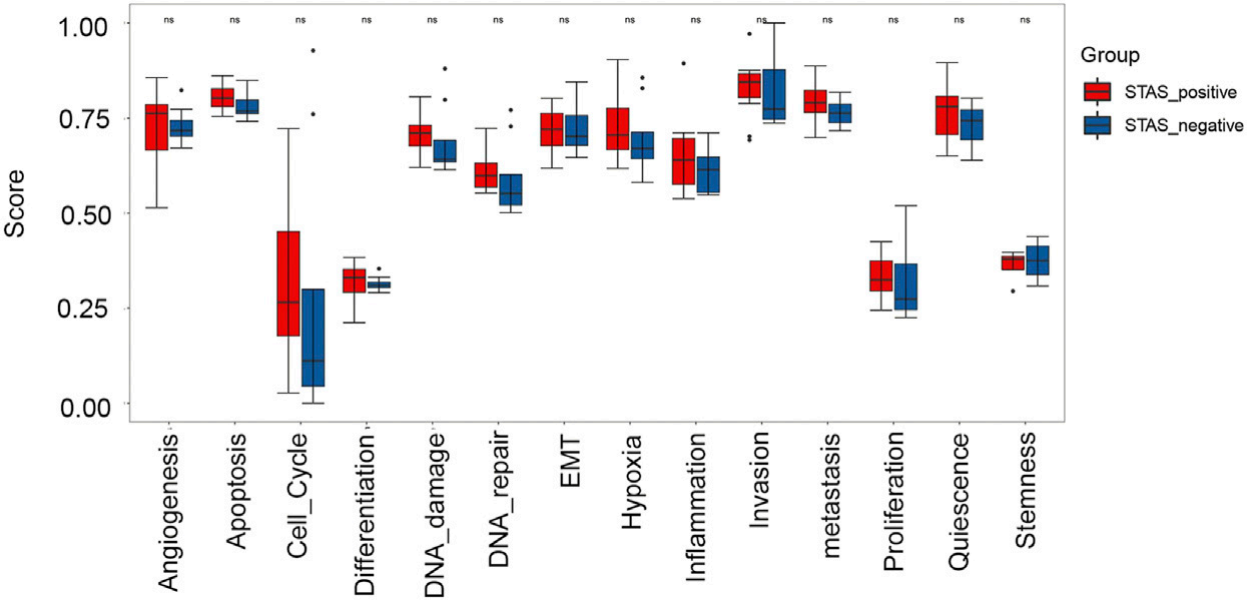
Box plot analysis of 14 functional status features between STAS_positive and STAS_negative groups.

Fourteen functional status features collected from the CancerSEA database were analyzed. Results exhibited that metastasis, hypoxia, DNA damage, proliferation, apoptosis, and cell cycle showed a trend to be higher in the STAS_positive group but failed to reach statistical significance (*p* > 0.05) (Figure 8A).

## Discussion

More than half of stage IA lung adenocarcinoma patients with STAS who receive radical surgery will relapse within 5 years (Onozato et al., 2013). Investigations have demonstrated that STAS occurrence is closely related to clinical outcomes, such as disease recurrence and survival rates. Furthermore, STAS is considered an independent prognostic factor for OS and DFS (Lu et al., 2017; Yanagawa et al., 2018; Aly et al., 2019). However, the current knowledge about markers and molecular pathogenesis of STAS in lung adenocarcinoma is limited. In our study, we performed a comprehensive bioinformatics analysis of gene expression in STAS_positive and STAS_negative samples and screened key genes that may be involved in the pathogenesis of STAS. We found that high CXCL8 and low CPB2 expressed in STAS_positive samples were clinically significant. In addition, high CXCL8 and low CPB2 expression levels were risk factors for unfavorable survival through analysis of TCGA. Consequently, we identified CXCL8 and CPB2 as valuable biomarkers for the diagnosis and prognosis of patients with STAS, and these may be promising therapeutic targets for the treatment of STAS as well.

CXCL8 is a chemokine that severely promotes neutrophilic inflammation through its receptors, CXCR1/CXCR2 (Ha et al., 2017). CXCR1 and CXCR2 are widely expressed on neutrophils, endothelial cells, cancer cells, and tumor-associated macrophages (Gregson et al., 2013). The CXCL8-CXCR1/2 axis plays a crucial part in tumor progression and metastasis by regulating the proliferation and self-renewal of cancer stem cells (Li et al., 2019). CXCL8 is overexpressed in many solid tumors, including lung, esophageal, breast, and colon tumors. Notably, CXCL8 binds CXCR1/2 in the tumor microenvironment, promoting tumor cell proliferation and growth through autocrine and paracrine mechanisms (Xie, 2001; Lin et al., 2004). Additionally, CXCL8 regulates hepatocellular carcinoma (HCC) cell proliferation and migration. The increased expression of CXCL8 in HCC cells accelerates tumor proliferation, migration, and invasion and is strongly correlated with clinical stage and tumor infiltration (Yang et al., 2020).

In addition to its regulatory properties, CXCL8 is a potential biomarker to predict tumor progression and prognosis in many malignancies. For example, in melanoma and breast cancer, CXCL8 overexpression plays a key role in metastasis and poor patient survival outcomes (Wu et al., 2012; Fang et al., 2017). In addition, higher expression of CXCL8 has been noted in ovarian cancer cell lines with high metastasis compared with the parental cell lines (Milliken et al., 2002). Similarly, CXCL8 promotes tumor growth and metastasis and predicts bad outcomes in colorectal cancer (Xiao et al., 2015). Therefore, these studies demonstrate an important role for CXCL8 in tumorigenesis and metastasis. In the present study, we found that high CXCL8 expression could promote the occurrence of STAS, which is consistent with previous studies noted above.

CPB2, also known as thrombin activatable fibrinolysis inhibitor (TAFI), plays a central role in coagulation and fibrinolysis (Mosnier and Bouma, 2006). It is understood that coagulation and fibrinolytic systems are correlated with physiologic and pathological processes such as tumor growth and invasion (Higuchi et al., 2009). Additionally, plasma CPB2 levels are positively associated with many human diseases (Boffa and Koschinsky, 2007). Similarly, increased expression of CPB2 has been reported in breast, ovarian, lung, gastric, and hepatic cancer cells (Yeung et al., 2013; Miller et al., 2011; Hataji et al., 2004; Fidan et al., 2012). Furthermore, tumors with high CPB2 expression have been related to more advanced tumor stages (Balcik et al., 2011). Conversely, it has been reported that downregulation of CPB2 expression by siRNA reduces breast cancer cell proliferation, migration, and invasion (Yu et al., 2017). Contrary to the findings of the above studies, our results showed that low expression of CPB2 may facilitate the appearance of STAS and be relevant to worse PFS in adenocarcinoma. At the same time, previous research in cell line models has demonstrated that CPB2 results in the suppression of breast cancer cell invasion and migration, which is consistent with our findings (Bazzi et al., 2016). The reason for this difference may be that there are fewer articles about the CPB2 gene. Therefore, the *CPB2* gene is worth exploring further to shed light on underlying mechanisms in cancer cells.

There are some noted limitations in our study. Firstly, a major limitation is the heterogeneity of the analyzed samples, so all significant results should be interpreted with caution. Secondly, multiple lines of evidence suggest that these predictions based on epigenetic profiling are largely accurate (Sharp et al., 2011). However, these computer-based predictions do not obligatorily reflect the actual gene pool and need to be verified experimentally in future work.

In conclusion, we revealed the potential markers and underlying molecular mechanisms of STAS in lung adenocarcinoma through a systematic and comprehensive analysis of the high-throughput sequencing RNA-seq dataset. Our results provided promising clues and laid the groundwork for developing new effective clinical therapies for STAS. However, further study is needed to better understand STAS and develop new treatments by integrating more data.

## Data Availability

The original contributions presented in the study are publicly available. These data can be found here: GSA database: HRA002248.
